# The Trained Nurse the Nation's Creditor

**Published:** 1918-06-08

**Authors:** 


### THE ECONOMICS OF NURSING.

KNUTSFORD.

### THE TRAINED NURSE THE NATION'S CREDITOR.

There are a variety of reasons, pressing and important, why the economics of nursing, or the principles and circumstances which govern nurses' employment, should be understood by the profession. One of these is that a call has been made to the nation to establish a Fund for British Nurses, and that a party of factionists within the fold are endeavouring by means of propaganda to demonstrate that this call is unauthorised, unjustified, and demeaning. Such a charge is either true or false. The issue is not speculative, not merely a matter of opinion. It is simply a case of debtor and creditor, and proof can be adduced as in commercial transactions.

There are several classes of trained nurses which, for convenience sake, may be arranged as follows:—(*a*) Government and municipal employees, including the nursing staffs of the Navy and Army; (*b*) private nurses, including employees in private nursing institutions; and (*c*) hospital nurses. It is with these last that I am now concerned.

In considering this subject it is necessary to bear in mind that a hospital is a public institution provided for the benefit of the nation's poor, and that it depends for its existence on funds supplied by the public, and on an adequate and efficient staff of physicians, surgeons, and nurses. With the medical staff I am not immediately concerned. In broad and general terms they owe as much to the hospital as the hospital owes to them. It is at once their training-school and their school of experience, but it is not their means of livelihood. The nurse

**THE ECONOMICS OF NURSING**—(*continued*).

is in a different category. She joins as a probationer, and if competent becomes a staff sister, and, ultimately, if she is particularly fortunate, a matron. The hospital is her alpha and omega. She devotes her entire time to her work, and her duties are more exacting than those in most other spheres of life, including even domestic service.

Now the hospital is not, nor is intended to be, a money-making institution. On the contrary, the majority are in a chronic condition of debt, and boards of management are often at their wits' end to find the necessary funds. The onus of what is a national obligation is borne by the generosity of a few, for the man in the street has not yet recognised that he is his brother's keeper. In these makeshift circumstances it would be unfair to offer any harsh criticism of hospital boards. They do their utmost to discharge what is often a thankless duty. The real sinner is the public, whose conscience has not hitherto been touched. The hospital is there, and few take the trouble to inquire how it came, or who pays the piper. A future generation will doubtless regard us with the same feeling of contemptuous pity with which we contemplate our ancestors who tolerated Dotheboys Hall and Sarah Gamp. We pride ourselves that we have eliminated both, but it is not even dimly realised that the hospital nurse is the nation's creditor. It is not admitted, even in hospital reports, that the nurse is the involuntary victim of mediævalism. Her name is not inscribed in the roll of contributors, but, nevertheless, she is the chief corner-stone of the edifice. She toils at half wages in the public service.

When a merchant embarks in commerce he has one sole object in view, and that object is profit. His salesmen and assistants are instruments of profit. The man or woman who fails to make a department "pay" is superseded, and without remorse. On the other hand, unqualified success is invariably rewarded. This is a generally recognised principle of commerce and accords with the ethics of our civilisation. The hospital nurse toils at a wage substantially below her market value. Her hours of labour are longer and more arduous than those of her fellows, equally educated and equipped, and her holidays are fewer. Scarcely ever has she any pension outlook. Lord Knutsford tells her that hers is a hard life, a life of sacrifice—but he does not explain why, nor does he encourage her to seek betterment. On the contrary, he deprecates any "growing tendency to growl" for improved conditions of employment.

Profiteering in Nurses.

I do not suppose it has ever occurred to Lord Knutsford that profiteering in nurses' labour, even in the noble cause of the destitute, is immoral. The distinguished Chairman of the London Hospital is in truth a philanthropist, and far be it from me to attempt to detract from the magnificent service that he has performed for the poor of the East End of the Metropolis. Moreover, I am willing to concede that as hospitals go the London Hospital is a model employer. Nevertheless I cannot evade the issue. From the figures published in the report I am bound to state that, so far as nurses are concerned, the London Hospital is in actual fact a sweating establishment. Here I find set out in plain terms the market value in the Metropolis of a trained nurse's services. Over two hundred are constantly employed by the London Hospital for attending on private patients, and the scale of charges ranges from two and a half to three and a half guineas per week, with board and lodging. The women who earn these fees receive £35 salary in their third and £40 in their fourth year of service. The remainder is the hospital's profit, and goes to swell the balance sheet.

The nurses on the private staff at the London, are, as in the case of the merchant's employees, instruments of profit; but inasmuch as the hospital does not supply the capital, which the merchant does, I submit that this profit is the nurse's property, confiscated willy-nilly.

I submit, furthermore, that what the hired-out nurse earns the ward nurse also earns. Her market value in the Metropolis does not depend on whether her services are availed of in a West End residence or in an institution for the poor.

It will, doubtless, be contended in reply that the nurse owes this tribute to the institution in return for her training. This is a commonly received doctrine. But it is a fallacy. The doctor equally owes his knowledge and skill to the hospital, but what doctor would consent, when his training is complete, to work for his upkeep, and allocate the balance to a charitable institution?

The moral is obvious. The hospital nurse is the nation's creditor, and every board of managers in the country owe it to their staffs to see that the National Fund is made adequate to meet the debt that it is intended to pay. Whatever its ultimate dimensions it will have been twice earned. The public conscience must be awakened, and the facts, of which they are entirely unaware, must be submitted for their consideration. If this is done with thoroughness and without reserve the British public will respond. IERNE.

